# Cognition of dairy cattle: Implications for animal welfare and dairy science

**DOI:** 10.3168/jdsc.2025-0860

**Published:** 2025-11-07

**Authors:** Kathryn L. Proudfoot, Thomas Ede, Catherine L. Ryan, Heather W. Neave

**Affiliations:** 1 Sir James Dunn Animal Welfare Centre, Atlantic Veterinary College, University of Prince Edward Island, PE, Canada, C1A4P3; 2 Department of Clinical Studies, University of Pennsylvania School of Veterinary Medicine, Kennett Square, PA 19104; 3 Department of Psychology, University of Prince Edward Island, PE, Canada, C1A4P3; 4 Department of Animal Sciences, Purdue University, West Lafayette, IN, 47907

## Abstract

The study of dairy cattle cognition has gained increasing attention over the past several decades, offering insights into the relationship between cognition and animal welfare. The objectives of this narrative review are to summarize a selection of studies exploring different cognitive processes in dairy cattle, discuss how these processes relate to common management practices and animal welfare, and identify knowledge gaps to guide future research. We begin with a brief overview of research into how dairy cattle perceive and sense the world around them, followed by a description of different types of learning and memory studied in dairy cattle, including nonassociative and associative learning, as well as short- and long-term memory. We then discuss how researchers have explored cognitive processes in dairy cows to understand their social lives, their ability to cope with challenges, and how they feel under different management conditions. Continued research into dairy cattle cognition is encouraged, including both foundational studies asking questions about the cognitive abilities of dairy cattle, as well as applied questions that can lead to improvements to their housing and management. We end by offering several avenues of future research into the cognition of dairy cattle, including a better understanding of competence and resilience, factors that influence cognition such as sleep and individual differences, as well as other under-investigated topics, such as problem-solving and metacognition.

## Highlights

• Dairy cattle cognition is a growing area of research with implications for welfare.
• Cattle show complex perception, learning, and memory.
• Cognitive and behavioral flexibility allows cows to adapt to different environments.
• Further research is encouraged to explore the role of dairy cow cognition in animal welfare.

## Graphical Abstract

**Figure:**
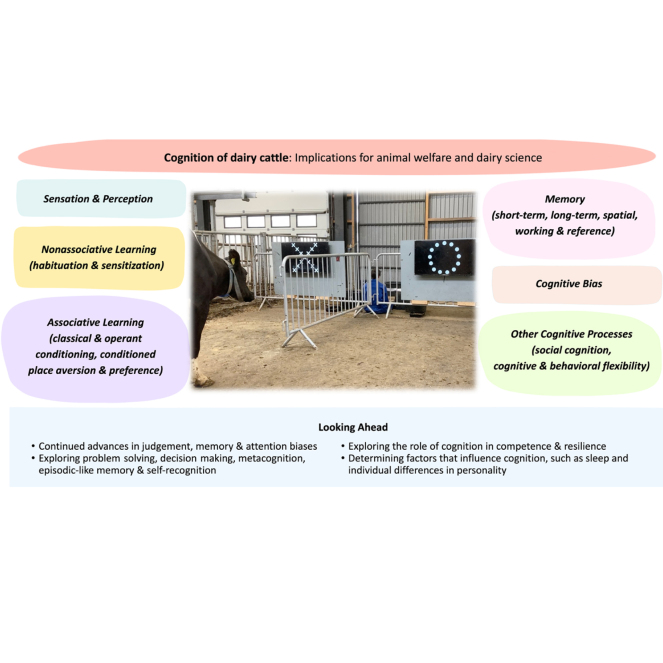
**Summary:** Understanding how dairy cattle perceive, retain, and respond to information from their caretakers and the environment has received growing interest from researchers over the past several decades. The aims of this review are to explore what we currently know about dairy cattle cognition and how it relates to their welfare, as well as highlight knowledge gaps to stimulate future research. Photo by Heather W. Neave and Margit Bak Jensen: Dairy cow in an active choice judgment bias task, Danish Cattle Research Centre, Aarhus University, Denmark.

The study of dairy cattle cognition has emerged as a growing area of scholarly interest over the last several decades (e.g., see reviews: R⊘rvang and Nawroth, 2021; Neave, 2025). Cognition, or the ability to “acquire, process, store, and act on information from the environment” (Shettleworth, 2010, p. 4), is an essential survival tool for animals, allowing them to make quick adaptations to their changing environments and circumstances. Recognizing and understanding the cognitive abilities of animals can provide insight into their emotional (affective) states, which is a core component of their welfare (Fraser et al., 1997). Franks (2018) proposes that the relationship between cognition and welfare is bidirectional: Cognition can affect animal welfare (e.g., providing cognitively enriching environments can improve affective states), and animal welfare can influence cognition (e.g., animals experiencing negative affective states may show hindered learning abilities).

To date, research on dairy cattle cognition has advanced our understanding of their cognitive abilities and revealed opportunities to improve their care (e.g., see detailed review by R⊘rvang and Nawroth, 2021). As research in this field continues to evolve, the goal of this short narrative review is to encourage further discussion and interdisciplinary research in dairy cattle cognition. The specific objectives are to summarize a selection of studies exploring different cognitive processes in dairy cattle, discuss how these processes relate to common management practices and animal welfare, as well as identify knowledge gaps to guide future research.

A starting point for understanding cognition in dairy cattle is how they perceive and sense the world around them. Knowledge about cattle sensation (e.g., the sensory input, including sight, smell, touch, taste, hearing) and perception (i.e., the brain's interpretation of that input) can help us design appropriate experiments, environments, and handling procedures. For example, dairy cattle can discriminate between features of their environment such as colors, shapes and smells (reviewed by R⊘rvang and Nawroth, 2021), which can be used to create effective paradigms to assess animal learning. In some cases, cows may fail to learn a task simply because we do not understand what they can and cannot perceive. This phenomenon is relevant across dairy science, as a cow's response to an experimental intervention may be affected by cues that the researcher may be unaware of (e.g., shadows, smells). Further research is encouraged to determine how basic sensation and perception in cattle can facilitate the design of environments and experiments that are meaningful, promote positive experiences, and minimize stress-inducing stimuli.

Researchers have also explored different types of learning and memory in dairy cattle and how these can provide insight into their welfare. For example, dairy cattle have demonstrated habituation, a basic form of nonassociative learning that does not rely on the ability to make associations between stimuli. Habituation occurs when an animal's response to a repeated stimulus decreases over time, as the animal learns that the stimulus is nonthreatening. For example, Kutzer et al. (2015) exposed heifers to milking procedures for 10 d before calving and found that, compared with control animals, the habituated heifers showed fewer steps and kicks after calving during milking, and many showed a decline in flight distance to human handlers.

Dairy cattle also experience the other form of nonassociative learning, sensitization*,* whereby animals may become more reactive to a repeated presentation of a stimulus, such as dairy cows being more sensitive to repeated loud noises at auction (Lanier et al., 2000). Predicting whether an animal will habituate or sensitize to a stimulus is difficult, but in general, animals tend to habituate when the stimulus is of low to moderate intensity and sensitize to more intense stimuli (Domjan, 2015). According to Domjan (2015), habituation and sensitization are both important mechanisms of emotional modulation; pleasure, fear, exploratory motivations, and aggression can all be modified by these fundamental learning mechanisms. Further studies on nonassociative learning in dairy cattle are encouraged to explain and reduce behavioral reactivity to new environments and handlers.

Dairy cattle also exhibit associative learning, such as classical (Pavlovian) and operant conditioning, which require cattle to make associations between 2 stimuli. For classical conditioning, an animal associates a neutral stimulus with a stimulus (an unconditioned stimulus) that naturally provokes a response (an unconditioned response). For example, in a study of virtual fences, Verdon et al. (2021) trained cows to associate a tone to a brief electrical pulse to provoke the cows not to enter a restricted area of pasture; cows soon learned to avoid the restricted areas after hearing the tone alone. Lee et al. (2018) suggest that virtual fences may provide greater predictability and control than traditional electric fences for animals that learn to associate the tone with the electric pulse. However, animals that fail to learn this association may face increased welfare risks due to reduced predictability and control. Although not well studied in dairy cattle, classical conditioning can also modify emotional, endocrine, and immunological responses in animals, such as taste-immune associative learning in rodents (reviewed by Hadamitzky et al., 2020). Thus, there remain several opportunities for further exploration of classical conditioning and its role in dairy cattle welfare.

For operant conditioning, the likelihood that a cow performs a specific behavior is either increased (reinforcement) or decreased (punishment) based on its consequences, such as adding a stimulus (positive) or removing a stimulus (negative). Research on this concept in dairy cattle has been mainly focused on positive reinforcement, such as training dairy cows to enter automated milking systems by rewarding them with grain (Jacobs and Siegford, 2012) and training heifers to stand still for an injection (Lomb et al., 2021). Positive reinforcement training may have several benefits to cattle, as it has been shown to elicit play behaviors in heifers, suggesting that the animals find the training itself rewarding (Heinsius et al., 2024), and may also play a role in more positive relationships between cattle and their caretakers. In contrast, although not well-studied in dairy cattle, researchers caution the use of punishment as a training tool; although punishment can be effective at stopping an unwanted behavior, it can also result in fear (Casey et al., 2021).

Researchers have also used paradigms of associative learning to more directly understand affective states in cattle. For example, conditioned place aversion tests have been used to determine if dairy cattle associate their surroundings with a negative experience. Ede et al. (2019) showed that dairy calves will avoid an area with visually distinct cues (red squares vs. blue triangles) if they had been associated with hot-iron disbudding compared with a sham procedure. Conditioned place aversion has been further applied to explore length of transportation (Creutzinger et al., 2022), social buffering (Nogues et al., 2023), and hunger (Lafon et al., 2024) in cattle. Both conditioned place aversion and its counterpart, conditioned place preference, are promising areas for further research in dairy cattle.

In addition to understanding how cattle acquire new information, researchers have also studied their memory (how they store, retain, and retrieve this information) to guide future behavior (Shettleworth, 2010). Researchers typically study one of 2 characteristics of cattle memory: what is remembered (e.g., a location), and the duration of the memory. For example, with sufficiently high initial food motivation and gradual learning, cows can solve a complex maze and remember it for up to 6 wk (Hirata et al., 2016). Calves previously loaded into trailers with the aid of sugar cubes were easier to load and showed fewer stress indicators 5 wk later (Fukasawa, 2012). Similarly, heifers exposed to brushing and haltering early in life were less reactive in tests of fearfulness involving humans, suggesting memory of at least 6 mo of positive handling (Boissy and Bouissou, 1988). A recent pilot study noted that heifers remembered multiple tasks, such as coming when called or touching a target, for up to a year after their training (de Boyer des Roches et al., 2025).

Cattle also display multifaceted spatial memory (i.e., using information from within and across trials), believed to improve foraging efficiency (Laca, 1998). This idea has been tested in calves by adapting the hole-board test using bottles baited with milk among empty bottles, assessing calves' working memory (avoiding milk bottles already visited during a test session) and their reference memory (learning to return to locations where food was found in previous sessions; Lecorps et al., 2022). Decreasing calves' milk allowance (from 12 L/d to 6 L/d) led to a decline in memory in the hole-board test, likely driven by feelings of hunger (Lecorps et al., 2023). Together, these studies on cattle memory demonstrate the complex and long-term memory of dairy cattle, the link between affective states and memory, and the value of building long-lasting positive relationships between humans and animals.

As herd animals, dairy cattle lead rich social lives that require them to recognize, adapt to, and learn from others in the group, collectively referred to as social cognition. Dairy cattle can discriminate between conspecifics (Suchon et al., 2025) and can tell the difference between individual cattle using their faces alone (Coulon et al., 2011). However, familiarity of the conspecific may affect speed of learning, and not all individuals learn to discriminate between unfamiliar conspecifics (Suchon et al., 2025).

Social learning, a component of social cognition, occurs when individuals acquire new behaviors or information by observing or interacting with others. This differs from social transmission, where conspecifics influence existing behaviors, such as through social facilitation (increased behavior in the presence of others) or local and stimulus enhancement (increased attention to certain objects or locations). Such processes can help naïve animals navigate novel environments more efficiently by relying on the experiences of others (Bandura, 1977). Both social transmission and social learning influence behavior in many farmed species, but evidence for social learning in cattle is limited. Only one study has directly tested this: Stenfelt et al. (2022) found that observer cows did not outperform controls that did not observe a demonstrator cow complete a spatial task, suggesting reliance on individual rather than socially acquired information.

Social cognition also extends to how cattle recognize, remember, and modify their responses to their human caretakers. For example, researchers have shown that cows are capable of distinguishing between people (Munksgaard et al., 1997; Rybarczyk et al., 2001). Although the specific cues that cattle use remain unclear, it is likely that a combination of clothing, faces, or height play a role. Munksgaard et al. (1997) found that cows can also associate specific people with aversive handling; cows kept a greater distance from people who had previously subjected them to negative handling (strikes on the head with open palm) compared with those who had previously provided positive interactions (food and petting). Cows have also been shown to remember people who handled them using aversive techniques. For example, Rushen et al. (1999) showed that cows had increased heart rate and more residual milk after milking when a person who had previously handled them negatively was present in the milking parlor compared with a person who had previously handled them gently. More research on the possible benefits of positive interactions between cattle and their caretakers is warranted.

Dairy cattle have also been shown to exhibit cognitive and behavioral flexibility, or the ability to adapt responses when environmental conditions change (Uddin, 2021). Cognitive flexibility refers to the mental capacity to adjust information processing or decision-making strategies, whereas behavioral flexibility describes the ability to modify actions accordingly. This flexibility is typically assessed using reversal learning tasks and intra- or extradimensional shifts (e.g., changes to previously learned T-shaped mazes). Reversal learning tasks have been used to evaluate how housing and management of dairy calves affect their cognitive functioning. For example, calves raised with their dams or peers performed better on a reversal task compared with individually housed calves, suggesting that social contact early in life improves cognitive flexibility and adaptability (Gaillard et al., 2014; Meagher et al., 2015). Similarly, dietary improvements, such as access to hay, improved calves' performance in a T-maze when the rewarded arm changed (reversal task) or when an object was unexpectedly placed in the rewarded arm (intramaze shift task; Horvath et al., 2017). Calves that succeeded in these types of tasks also showed smoother integration into new social groups after weaning, highlighting how cognitive flexibility may lead to improved social adaptability (Horvath and Miller-Cushon, 2018).

Other cognitive processes that have received little or no attention in cattle include problem solving and decision making. Tasks that test for these processes have been developed for other farm animals, which could be drawn upon for application in dairy cattle. For example, the Pig Gambling Task has pigs choose between small, more frequent rewards versus larger but less frequent rewards, which tests cognitive and emotional processes related to decision making, impulse control and risk assessment (van der Staay et al., 2017). “Impossible” tasks have been used to investigate how animals respond when a previously solvable task becomes unsolvable; animals must first learn to solve the task, but the test trial assesses their subsequent behavior (e.g., attention to or interaction with the human experimenter) once the solution is no longer possible. In farm animals, this task has often focused on human–animal communication or social referencing rather than problem solving itself (e.g., in goats; Langbein et al., 2018). Detour tasks also test an animal's problem-solving ability and inhibitory control by needing to navigate around a transparent or opaque barrier (i.e., temporarily move away from the reward) to reach a visible reward (Nawroth et al., 2016), with only one such test performed in dairy cows to our knowledge (Stenfelt et al., 2022). Adapting tasks that have been developed in other species will allow us to better understand these abilities in dairy cattle and their implications for welfare.

Researchers are increasingly recognizing that an individual's cognitive function is heavily influenced by their affective state; thus, measuring differences or changes in cognitive function can be a way of indirectly evaluating how the animal feels under different management conditions. In the past decade, cognitive bias tasks have emerged as a promising method to evaluate how emotion influences information processing (Mendl et al., 2009). The most common approach, judgment bias, measures how animals interpret ambiguous cues by training them to associate one cue with a positive outcome (e.g., a reward) and another with a negative outcome (e.g., no reward or a mild punishment). Once trained, animals are presented with ambiguous, intermediate cues to assess whether they respond as if expecting the positive or negative outcome; these responses are interpreted as optimistic or pessimistic, and positive or negative emotional states, respectively.

Cognitive bias paradigms have been applied to understand the affective states of dairy cattle under different conditions. For example, calves were pessimistic following disbudding (Neave et al., 2013) and dam separation (Daros et al., 2014), suggestive of a negative emotional state, and were optimistic when pair-housed versus individually housed (Bučková et al., 2019), suggesting a positive emotional state. However, cows did not show the expected optimism when housed on pasture (Crump et al., 2021) or an enriched indoor housing (Kremer et al., 2021) despite being motivated for these conditions. Attention bias (differential attention toward threatening or rewarding stimuli, depending on emotional state) has also been explored in dairy cows with mixed results (Franchi et al., 2020; Kremer et al., 2021). However, memory bias (differential recall of positive and negative memories, depending on emotional state; see Burman and Mendl, 2018) has never been explored in dairy cattle to date. In general, cognitive bias tests offer valuable insight into affective states by drawing upon an understanding of how emotions affect the way we think and process information.

As the field of animal welfare continues to evolve, there is a growing need for further applied and basic scholarly work that advances our knowledge about the cognitive abilities of dairy cattle and enhances our understanding of their welfare. For example, cognitive approaches have the potential to play a pivotal role in advancing the emerging field of positive animal welfare, which recognizes that the promotion of positive experiences, rather than merely mitigating negative ones, is critical for an animal to have a good life (Rault et al., 2025). Within this framework, researchers are currently exploring effective ways to study animal competence (i.e., their ability to effectively interact with and respond to their environment) and resilience (i.e., their ability to maintain or recover mental and physical functioning when faced with challenges). Continued research into cognitive and behavioral flexibility, as well as problem solving and decision making, will provide important insights into how dairy cattle learn to navigate complex aspects of their environment across different life stages.

Research is also needed to understand factors that influence cognition in dairy cattle, such as sleep and individual differences in personality. For humans and laboratory animals, researchers have shown that sleep organizes newly acquired information, consolidates memories, and improves attention, learning, habituation to emotional stimuli, cognitive flexibility, and problem solving (Palmer et al., 2024). Although we understand some basics about sleep in dairy cattle (Proudfoot and Ternman, 2024), research is encouraged to explore how sleep and sleep loss affect cognitive performance in cattle. Moreover, exploring individual differences in processing information and solving problems is another key avenue for future work. There is evidence that individual cows develop different strategies for cognitive tasks, including basic processes such as habituation (Paranhos da Costa et al., 2021). Identifying and linking cognitive strategies to personality in cattle will also help us build a more comprehensive view of how individuals cope with challenging environments.

The research to date has scratched the surface of understanding the cognitive abilities of dairy cattle. A further step is the exploration of more complex processes, such as metacognition (thinking about one's own thinking), episodic-like memory (recalling the “what,” “when,” and “how” of an experience), or self-recognition (the ability to recognize one's own self). Each of these processes are experimentally and theoretically challenging but have been explored in other mammalian species (e.g., Crystal, 2010). Evidence of such processes in nonhuman animals has shifted scientific and ethical perspectives on animal minds, highlighting the need for environments and management practices that reflect and support their cognitive and emotional capabilities (Nawroth et al., 2019). These emerging areas of inquiry have the potential to deepen our understanding of how dairy cattle experience their own learning, recall and emotionally process past experiences, and respond to the affective states of conspecifics. Future work might combine behavioral approaches with novel techniques, such as noninvasive neuroimaging, electrophysiological measures, or pharmacological approaches, to investigate these complex cognitive processes in cattle.

Overall, current research demonstrates that dairy cattle possess complex cognitive abilities, including sophisticated learning, memory retention, as well as cognitive and behavioral flexibility. Although we have much more to learn, recognizing and understanding these abilities has important implications for anyone who works with dairy cattle, including the farmers who care for them, dairy scientists using them in their research, and animal welfare scientists seeking to improve their welfare.
